# Supplementary material for: Testing and Validation of a Custom Retrained Large Language Model for the Supportive Care of HN Patients with External Knowledge Base
Source: Cancers (Basel). 2024 Jun 24;16(13):2311. doi: 10.3390/cancers16132311 (PMC11240646; doi:10.3390/cancers16132311)
Supplement: Supplementary file 1 [file cancers-16-02311-s001.zip › cancers-3050764-supplementary.pdf]

This supplementary material includes 20 questions and retrained LLM's responses. Actual patient questions are Questions 1-10. ChatGPT-4 generated questions are Questions 11-20 on Xerostomia, Dysgeusia, Dysphagia, Fatigue, Anxiety, Lymphedema, Dental Issues, Dehydration, Mucositis, and Nausea.

Question1:

I think we need to ask Dr. xxx for an antibiotic or something. I believe I'm developing tracheitis, I think? My stoma inside on top is getting painful and the same blood results. Dr. xxx said its common for the stoma to be sore from radiation. He said it's from radiation and I should reach out to you. I'm just getting frustrated it hurts to wear to Lary button, and I can't wear sensitive adhesive because I can't use lotion there. What should I do?

Epic Answer: Yes, this sounds very frustrating. Sometimes this type of radiation side effect to the stoma and peristomal region can temporarily preclude a patient from wearing a baseplate or button. First, I would see the ENT PA (who can communicate with Dr. xxx) in case you can't get in right away to see Dr. xxx - to let them render medical advice about what they see with regards to the wound and what they would recommend for addressing it. From a stomal housing standpoint, I think it may be best to (again, temporarily) go back to the Lary TUBE (so that you can wear the HME 24/7) but that way it's not putting pressure on the stoma ridge. It probably would be more comfortable.

AI Answer: Based on the information provided, it seems you're experiencing discomfort and pain due to radiation effects. It's important to manage these symptoms effectively. For the pain, you could potentially use a prescribed medication like viscous lidocaine, which can be used for mouth and throat pain. For the issue with the Lary button, you might want to discuss with your doctor about alternative options or adjustments that can be made to make it more comfortable. It's also important to keep your doctor informed about your symptoms, as they can provide the most appropriate advice and treatment based on your specific condition. If the pain persists or worsens, it would be advisable to seek immediate medical attention.

Question2:

My neck started getting puffy with fluid from my jaw line/chin down to the middle of my neck, the area that was treated. It doesn't hurt but I'm getting a fat neck and I wonder if I should be concerned? Will it go away? It just started a week ago.

Epic Answer: This sounds like lymphedema, which is very common following radiation. xxx has placed an order for you to be assessed in the clinic and then she can make appropriate referrals from there. Our schedulers should be in contact with you soon.

AI Answer: The swelling you're experiencing could be lymphedema, a condition that can occur after radiation treatment. It's characterized by swelling due to a blockage in the lymphatic system, which prevents lymph fluid from draining well, leading to swelling. It's recommended to consult with a lymphedema specialist, usually a Physical Therapist or SLP with specialty lymphedema certification. They can assess your condition, show you how to massage the area, and recommend specialty compressive gear. The best approach for treatment is known as complete decongestive therapy which includes massage, compressive gear, and the pneumatic device on a daily basis. However, it's important to get this checked by your healthcare provider to confirm the cause and appropriate treatment.

Question3:

Patient is using the toothpaste above and all of a sudden it is burning his mouth when brushing his teeth. Any suggestions please advise. Hope all is well.

Epic Answer: The toothpaste can be put on hold until the mouth soreness heals. I recommend continuing the baking soda mouth rinses frequently throughout the day, and using viscous lidocaine as directed on bottle for pain.

If you need additional assistance, let me know a good time to give you a call.
AI Answer: The burning sensation could be due to a reaction to an ingredient in the toothpaste or a symptom of an underlying oral condition. It's recommended to stop using the toothpaste immediately and switch to a mild, non-flavored toothpaste. If the burning sensation persists, it would be advisable to seek an evaluation from a healthcare professional. It's also important to maintain good oral hygiene practices, such as rinsing the mouth with a saltwater solution and avoiding foods and drinks that are too hot, cold, spicy, or acidic, as they may irritate the mouth. Please note that this advice is based on the information provided and may not be applicable if there are other underlying conditions or symptoms not mentioned in your query.

Question4:
I'm having trouble with extreme dry mouth and the mucusitus is gaging me and really uncomfortable in the back of my throat. I have been drinking plenty of water
Epic Answer: Do you have any open sores in your mouth? Are there any painful areas?
If you haven't tried it already, you can try over the counter Biotene (it can be found in the oral care area in grocery stores) to help with dry mouth.
AI Answer: You can manage dry mouth by sipping water throughout the day, chewing gum or sucking on hard candy, frozen fruit, popsicles, and ice chips to stimulate saliva production. Avoid alcohol, drinks with caffeine, and acidic juices as they can make your mouth even drier. You can also use a cool mist humidifier, especially at night. For the mucus, Guaifenesin syrup can help dry up secretions. If the problem persists, it's recommended to consult with your healthcare provider. They may prescribe medications that stimulate saliva production or suggest other treatments.

Question5:
Good morning Dr.xxx, Since my prescription of Fluconazole has run out, I've noticed an increase in mouth sores, tongue bleeding and blood clots. I am wondering if refilling this medication is an option. Thank you.
Epic Answer: Our recommendation is that you be evaluated locally by either same day care or primary care provider. This will allow for in person examination of your mouth and tongue. As I mentioned earlier, they may decide to do a culture of the tongue.
To help with their assessment:
Radiation therapy completed on 8/11/22.
Fluconazole prescribed on 7/25/22 and again 8/18/22.
Management of side effects from radiation treatment will take time to improve. If you need further assistance, please let us know how we can help.
AI answer: I'm sorry to hear about your symptoms. Fluconazole is often used to treat fungal infections in the mouth, which could be causing your symptoms. However, it's important to evaluate your condition before refilling the medication. I recommend coming into our Immediate Care Clinic for an evaluation. If the sores are not due to a fungal infection, we can try a mouthwash mixture to help with the pain. Please seek immediate medical attention if your symptoms worsen.

Question6:
For some reason I am now having terrible nausea. I am even having difficulty taking water. Can you prescribe me some nausea medicine? RN spoke with patient and wife xxx over the phone. Per patient nausea started 2 days ago and unable to keep any fluids down, spouse stated that he "might have a cold." Patient taking Zyprexa and Zofran as recommended above with no relief.
Epic Answer: he can increase his Zyprexa to 7.5 mg PO QHS or add a 2.5 mg Zyprexa dose in am and continue the 5 mg at bedtime. If he truly has a "cold" or URI, there could be an infectious component to his

worsening symptoms as well. If he develops fevers/chills, dehydration from poor intake, he should be evaluated by a provider close to his home.

AI Answer: Based on the information provided, it's recommended to consider antiemetic agents for patients with persistent nausea. Prochlorperazine, metoclopramide, or haloperidol can be considered. If nausea persists, antiemetics should be administered around the clock for 1 week, then changed as needed. Other agents to consider include serotonin antagonists like Ondansetron, Granisetron, or FDA-approved cannabinoids such as dronabinol. If nausea persists after a trial of several opioids and above measures, reassess the cause and severity of nausea. However, it's important to consult with your healthcare provider for the best treatment option.

Question7:

So I have a quick question for you... I am not sure if I was wearing my Head and Neck bra too tightly last night, or if I slept awkwardly, but I woke up with some serious jaw pain on my right side (surgery side) this morning. It seems to have lessened, but I do have a bit of an ear ache in my right ear. It may be nothing, and my 3-month post radiation scans were clean (yay!) But I thought I would check with you. I definitely seem to be having allergies also which may be the culprit, but I promised I would ask you if I had any concerns/questions! :) Thoughts?

Thanks so much for your time and for always being so gracious and patient with me! :)

The pain has subsided. I do still have a bit of pain in my ear but it seems to be lessening. And my jaw isn't nearly as tight and painful as yesterday. Maybe it is allergies or a cold? Thoughts?

Epic Answer: Per xxx, it could be eustachian tube dysfunction caused by allergies. It could also be musculoskeletal or related to the compression device. Its hard to say, but we are glad you are feeling better.

AI Answer: Based on the information provided, it's possible that your discomfort could be due to wearing the Head and Neck bra too tightly or sleeping in an awkward position. However, it could also be related to allergies or a cold, as you suggested. If the pain has lessened and continues to do so, it's likely not a serious issue. However, if the pain persists or worsens, it would be advisable to seek medical attention. It's always a good idea to report any new or concerning symptoms to your healthcare provider, as you've done. Keep monitoring your symptoms and don't hesitate to reach out if you have further concerns.

Question8:

In the past 3 weeks I have been experiencing nerve tingling on the left side of my neck below my ear when I take a bite of food. It does not happen every time but it is of concern to me as this symptom is similar to what I experienced prior to being diagnosed with Cancer March 2021. I would like to know if it may be necessary to have another PET scan to verify if there is an issue and catch it early.

It is in the same or similar location where the Cancer was most prevalent.

It is not painful.

I do not have any swelling in that area of concern.

Epic Answer: Dr. xxx's team recommends that you are evaluated by Dr. xxx first before ordering any imaging. The physical exam will help Dr. xxx determine the appropriate exam to order.

AI Answer: Given your history of cancer and the recurrence of symptoms similar to those you experienced prior to your diagnosis, it is advisable to consult with your healthcare provider. They may recommend further evaluation, which could include imaging tests like a PET scan, to check for signs of cancer recurrence or treatment-related side effects. It's important to report any new symptoms to your doctor right away, as early detection can make a significant difference in treatment outcomes. Please reach out to your healthcare team to discuss these symptoms and the possibility of further testing.

Question9:
Hi xxx, is there anything that will stop or slow down mucositis? I've tried Mucinex but it didn't seem to help much. Any suggestions? Thank you.
Epic Answer: The side effects from treatment typically peak 10-14 days after treatment is completed. You should slowly start to see healing of your skin and mouth over the next 6 weeks.
Mucinex is helpful for managing secretions. Are you taking the prescription for Fluconazole as prescribed for thrush? For mouth sores and mouth pain, I recommend using viscous lidocaine, or the pain medications you have been prescribed.
I see you have a video follow-up with one of nurse practitioners this afternoon. I'm not sure if you have met xxx yet. She has been in our department for a long time and will be able to offer additional recommendations when you meet.
I am glad you have been able to return home as I'm sure your own environment is comforting to you.
AI Answer: Mucositis can be managed through a combination of good oral hygiene and specific treatments. Cleaning your mouth every 4 hours and at bedtime can help, using a soft-bristle toothbrush and mild rinses or plain water. If mucositis sores begin to crust over, a rinse of three percent hydrogen peroxide mixed with an equal amount of water or saltwater can be used for no more than 2 days.
Topical medicines for pain can also be used. If these are not effective, painkillers may be considered.
In terms of specific treatments, a study found that oral cryotherapy may help prevent mucositis and pain among patients undergoing chemotherapy. Another study suggested that gabapentin could be used for pain related to radiation-induced mucositis.
However, it's important to consult with your healthcare provider before starting any new treatments.

Question10:
If the ibuprofen is too much what should I do for pain? I literally have no voice. I stopped taking the Diflucan last night because at this point pain management is more important. How long does it take to get out of your system? I am completely miserable and can't take it anymore
Epic Answer: Are you tolerating the oxycodone okay? It looks like you has Perco cet 5/325 tablets and has been taking 1 every 6 hours. You can increase this to 2 tablets every 6 hours. I see that she has Xanax on her med list so I would encourage you not to take in combination is a can amplify affective be dangerous. you should go to your local ER for IV fluids or you can come here.
AI Answer: If ibuprofen is causing discomfort, you could consider increasing your dose of oxycodone. As per the information in the context, you have been taking Percocet 5/325 tablets every 6 hours. You can increase this to 2 tablets every 6 hours. However, it's important not to combine this with Xanax as it can amplify the effects and be dangerous. As for Diflucan, it generally takes about 5 to 7 days to get out of your system, but this can vary depending on individual factors such as your age, overall health, and other medications you may be taking. Please consult with your healthcare provider for personalized advice.

Question 11:
Why is my mouth so dry since I started my radiation? Is this going to go away or what?
AI: Radiation therapy can cause dry mouth by damaging the salivary glands, especially when the therapy is directed at the head, face, or neck. The amount of saliva made by the salivary glands usually starts to decrease within 1 week after starting radiation therapy and continues to decrease as treatment goes on. It can take 6 months or longer after radiation therapy ends for the salivary glands to start producing saliva again. Dry mouth often improves during the first year after radiation treatment, but many people continue to have some level of long-term dry mouth. Salivary glands may partly recover during the first year after radiation therapy, however, recovery is usually not complete, especially if the salivary glands received direct radiation.

Question 12:

Why does everything taste like cardboard since my treatments started? Is this going to last forever?

AI: The taste changes you're experiencing are a common side effect of cancer treatments like chemotherapy and radiation therapy. These changes can be due to damage to the taste buds, dry mouth, infection, or dental problems. Certain chemotherapy drugs and radiation therapy to the head or neck can harm the taste buds and salivary glands, causing taste changes.

In most patients receiving chemotherapy, taste returns to normal a few months after treatment ends. For radiation therapy, taste changes usually start to improve 3 weeks to 2 months after treatment ends and may continue to improve for about a year. However, if the salivary glands are harmed, the sense of taste may not fully return to the way it was before treatment.

It's important to communicate these changes to your healthcare team as there are ways to manage these symptoms and improve your nutritional intake during this period.

Question 13:

Why is swallowing food so hard all of a sudden? Is this going to get any better?

AI: The difficulty in swallowing could be due to various reasons. It could be a side effect of cancer treatment, oral mucositis, dry mouth, skin damage from radiation, infections, and graft-versus-host-disease (GVHD). It's also possible that the swelling in your neck and throat, as mentioned in your message, is contributing to the difficulty in swallowing.

As for whether it will get better, it often depends on the underlying cause. If the difficulty in swallowing is due to temporary side effects of treatment, it may improve once the treatment is completed. However, if it's due to permanent changes or damage, it might persist. It's important to communicate these symptoms to your healthcare provider, as they can provide specific advice and treatment options based on your individual situation.

Question 14:

Why am I so tired all the time since starting treatment? Is this the new normal for me?

AI: Cancer-related fatigue is a common side effect of cancer treatments such as chemotherapy, radiation therapy, and biological therapy. It can also occur after surgery and may last for months or even years after diagnosis. This fatigue can be a symptom of the cancer itself or a sign that the cancer is growing or spreading. Other health conditions, such as heart problems, hormone problems, arthritis, or nerve problems, can also contribute to fatigue. Your healthcare provider may screen for fatigue regularly using fatigue screening tools, which consist of a brief scale or survey. If you have moderate or severe fatigue months after initial treatment, or if fatigue starts or worsens after treatment ends, your provider will perform an in-depth assessment. It's important to communicate with your healthcare provider about your fatigue so they can help manage it effectively.

Question 15:

Why am I feeling like a bundle of nerves all the time since I got sick? Is this going to be my new thing?

AI: Feeling anxious or nervous is common for patients with a serious illness like cancer. This anxiety can be triggered by various factors such as finding a lump, receiving a diagnosis, fear of treatment, doctor visits, and tests. Signs of anxiety include uncontrolled worry, trouble focusing thoughts, muscle tension, restlessness, dry mouth, and irritability. It's important to recognize these feelings and take steps to manage them. If these symptoms interfere with your daily activities, a referral for mental health evaluation could be helpful. Therapy, sharing feelings, and joining support groups can often help manage these feelings. However, if you're experiencing severe distress, it's crucial to seek professional

help. It's normal to feel this way when you're sick, but with proper management, it's not necessarily a permanent condition.

Question 16:
Why's my arm (or leg) all swollen up since my surgery? Is there a way to get the swelling down?
AI: The swelling in your arm or leg post-surgery could be due to lymphedema, a condition caused by a blockage in the lymphatic system, part of the immune and circulatory systems. Symptoms include swelling, a heavy feeling, discomfort or pain, and skin changes. To manage lymphedema, it's recommended to maintain a healthy weight, avoid sitting or standing for long periods, wear loose-fitting clothes, limit time in extreme heat or cold, and seek medical care if you notice signs of infection. Regular exercise, good skin care, and wearing well-fitted compression garments can also help. If your arm or leg starts to ache, elevate it above the level of your heart. You may also benefit from seeing a certified lymphedema therapist. However, it's important to consult your healthcare provider for personalized advice.

Question 17:
My gums are so sore all the time now. Is this because of my treatment or something I'm doing wrong?
AI: Your sore gums could be a side effect of your cancer treatment. Radiation therapy to the head or neck, chemotherapy, and immunotherapy can cause mouth sores and tender gums. Dental problems or mouth infections can also make your mouth sore. It's important to maintain good oral hygiene during treatment. Gently brush your teeth twice a day, floss regularly, and avoid alcohol and extreme textures and flavors in your diet. If your gums continue to be sore, it's important to inform your doctor or dentist. They can help determine if the soreness is due to treatment or dental problems.

Question 18:
I've been feeling super thirsty lately, even more after my last treatment. Is this normal?
AI: The context provided does not directly address the issue of increased thirst following treatment. However, it's important to note that certain treatments can cause dehydration, which may lead to increased thirst. It's recommended to increase fluid intake, as discussed in. If the thirst persists or is accompanied by other symptoms, it's advisable to seek medical attention.

Question 19:
Why is my mouth so sore and raw since I started my treatment? Is this going to stop anytime soon?
AI: The soreness and rawness in your mouth are likely due to your cancer treatment. Treatments such as chemotherapy, immunotherapy, and radiation therapy to the head or neck can harm the fast-growing cells in the lining of your mouth and lips, causing mouth sores and tender gums. This condition is known as mucositis. Dental problems or mouth infections, such as thrush, can also make your mouth sore. The good news is that your mouth and gums will most likely feel better once the cancer treatment ends. If you're undergoing chemotherapy, the salivary glands usually return to normal after it ends. If the treatment is radiation therapy, the salivary glands may partly recover during the first year after therapy, but the recovery is usually not complete. It's important to communicate these side effects to your healthcare team as they can provide treatments to manage the discomfort.

Question 20:
Why do I feel like throwing up all the time since my treatment started? Is this going to stop?
AI: The feeling of nausea or vomiting can be a side effect of cancer treatments such as chemotherapy, radiation therapy, or immunotherapy. These treatments can harm healthy cells in your digestive tract, leading to these symptoms. The nausea or vomiting can occur right after treatment or 1 or 2 days later.

The type of cancer treatment you get and how you react to it affects how long you need to take these medicines. It's important to communicate with your doctor or nurse about these symptoms, as they can prescribe medicines to prevent or control vomiting (antiemetics or antinausea medicines). If the medicine is not helping, your doctor may prescribe another. The symptoms should improve once your treatment ends.
